# Development and initiation of a preceptor program to improve midwifery and nursing clinical education in sub-saharan Africa: protocol for a mixed methods study

**DOI:** 10.1186/s12912-024-02036-2

**Published:** 2024-05-31

**Authors:** Brittney van de Water, Kelsey Renning, Anda Nyondo, Mustapha Sonnie, Ashley H. Longacre, Helen Ewing, Mary Fullah, Lignet Chepuka, Julie Mann

**Affiliations:** 1 Seed Global Health, 20 Ashburton Place, 02108 Boston, MA USA; 2 Boston College, Connell School of Nursing, Chestnut Hill, MA USA; 3 Seed Global Health, Blantyre, Malawi; 4 Duke University, School of Nursing, Durham, NC USA; 5 Seed Global Health, Lilongwe, Malawi; 6 Seed Global Health, Freetown, Sierra Leone; 7 Ministry of Health and Sanitation, Freetown, Sierra Leone; 8 Kamuzu University of Health Sciences, Blantyre, Malawi; 9 Mount Auburn Hospital, Cambridge, MA USA

**Keywords:** Implementation science, Low-dose high-frequency training, Clinical learning environment, Mentorship, Capacity building, Program development, Training of trainers, Maternal and child health

## Abstract

**Background:**

Expanding the quality and quantity of midwifery and nursing clinical preceptors is a critical need in many sub-Saharan educational settings to strengthen students’ clinical learning outcomes, and ultimately to improve maternal and child mortality. Therefore, this study protocol was developed to establish a year-long, four step, precepting program to (1) improve partnership building and program development (2) provide an evidence-based course to expand competency and confidence in precepting students (3) select preceptors to become train the trainers and (4) secure accreditation for the program, ultimately to engage and support cohorts of preceptors and continue to monitor and evaluate the effectiveness of the program over five years.

**Methods:**

Qualitative and quantitative approaches will be used including evaluation of preceptors, faculty and leadership involved with the program, as well as students taught by preceptors. Data will include validated self-assessment scales, objective structured clinical examinations (OSCEs), satisfaction surveys, and direct clinical observation, in-depth interviews and/or focus group discussions (preceptors); feedback forms (students); process mapping and organizational readiness for implementing change surveys (faculty and leadership). Median change in scores will be the primary outcome for quantitative data. Content analysis within a deductive framework to identify key implementation and adoption themes will be used for qualitative data analysis.

**Discussion:**

This study aims to assess the readiness and early effectiveness for implementing a preceptor program for midwifery and nursing in Sierra Leone and Malawi. Determining the effectiveness of this program will guide future adaptations in order to strengthen the program for sustainability and potential scale-up.

**Supplementary Information:**

The online version contains supplementary material available at 10.1186/s12912-024-02036-2.

## Background

Countries in sub-Saharan Africa (SSA) remain with some of the highest maternal and child mortality rates in the world [1–3]. Specifically, Sierra Leone, West Africa has the world’s highest incidence of infant mortality (82 deaths per 1,000 live births) and the third highest incidence of maternal mortality (443 deaths per 100,000 live births) [1, 4, 5]. Malawi, in Southern Africa, also has an infant mortality rate of 31 deaths per 1,000 live births and an under-five mortality rate of 42 deaths per 1,000 live births [6]. Although midwives and nurses can be educated and equipped with the knowledge and skills to be primary care providers to pregnant people and children, there remains a shortage of highly qualified practitioners. The greatest shortage is in SSA, where Seed Global Health (Seed), an international non-government organization working to strengthen the healthcare workforce in sub-Saharan Africa, is focused [7, 8]. To address the shortage and increase coverage of midwife- and nurse-delivered interventions, the United Nations Population Fund (UNPF), World Health Organization (WHO), International Confederation of Midwives (ICM), and International Council of Nurses (ICN) call for bold investments in midwifery and nursing education and training on the global and local scale [8–10]. 

Recognizing the importance of clinical education in midwifery and nursing education, Seed Global Health undertook needs assessments in both Sierra Leone and Malawi to understand next steps for improving education [11–13]. The results of both assessments show the need for expanded quality and quantity of clinical preceptors to strengthen students’ clinical learning outcomes, ultimately to improve maternal and child mortality. Additionally, Seed Global Health has had longstanding partnerships in both countries, and specifically in Sierra Leone, was asked by the Ministry of Health and Sanitation to support strengthening of midwifery, as it has been prioritized in their National Nursing and Midwifery Strategic Plan 2019-2023 [7]. Preceptor programs have shown effective ways to promote critical thinking and advance learning of clinical concepts such as in Ghana, Pakistan, as well as improving patient outcomes in the USA, Ghana, and Kenya [14–17]. Additionally, large scale multi-country, multi-year programs, such as the U.S. President’s Emergency Plan for AIDS Relief, Nurse Education Partnership Initiative (NEPI) created high-quality, innovative, and sustainable nursing education models and resources for precepting [18]. However, the ongoing need for large cohorts of well-trained nurses and midwives are continually needed, especially with the skills to teach at the bedside.

Three key components to graduating competent nurses and midwives are: schools, clinical sites, and preceptors [19, 20]. Midwifery and nursing schools must prepare students for clinical practice and develop collaborative partnerships with clinical sites. Clinical sites need to provide an enabling environment for clinicians to offer - and model - quality care and hands-on learning opportunities for students. Finally, preceptors must be both clinically competent as well as knowledgeable in competency-based education to guide students’ clinical learning. Therefore, this study protocol was developed to establish a precepting program for midwives and nurses in four steps: (1) improve partnership building and program development (2) provide an evidence-based preceptor course to expand competency and confidence in precepting students in the clinical setting (3) select preceptors to become train the trainers for sustainability and scalability and (4) secure accreditation for the preceptor program. The goal of this program is to engage and support cohorts of preceptors previously trained, and continue to monitor and evaluate effectiveness of the program over five years.

## Methods/design

### Study design

The study design will incorporate qualitative and quantitative approaches to answer the research questions. The RE-AIM implementation science framework was chosen as the theoretical framework as well as the organizational readiness for change theory [21, 22]. This preceptor program utilizes a model of low dose, high frequency training as well as mentoring and role modeling. Implementation science aims to improve the quality and effectiveness of health services, and the use of high frequency, low dose training has been shown to be effective in midwifery and nursing education [23, 24]. Specifically, we will evaluate the organizational readiness to *implement* and *maintain* a comprehensive preceptor program within two Sierra Leonean midwifery institutions, and a Malawian nursing institution, specific to pediatric critical care. Additionally, we will determine the *effectiveness* of the preceptor program on preceptors’ competence and confidence as well as assess the *adoption* and *reach* of the preceptor program. The RE-AIM framework helps characterize the process and outcomes of planning and evaluating health programs [21]. This framework will guide and help capture the implementation of the preceptor program as part of improving maternal and infant health outcomes in Sierra Leone and Malawi through educating the next generation of midwives and nurses.

### Participants and recruitment

#### Preceptors

**Fig. 1:**
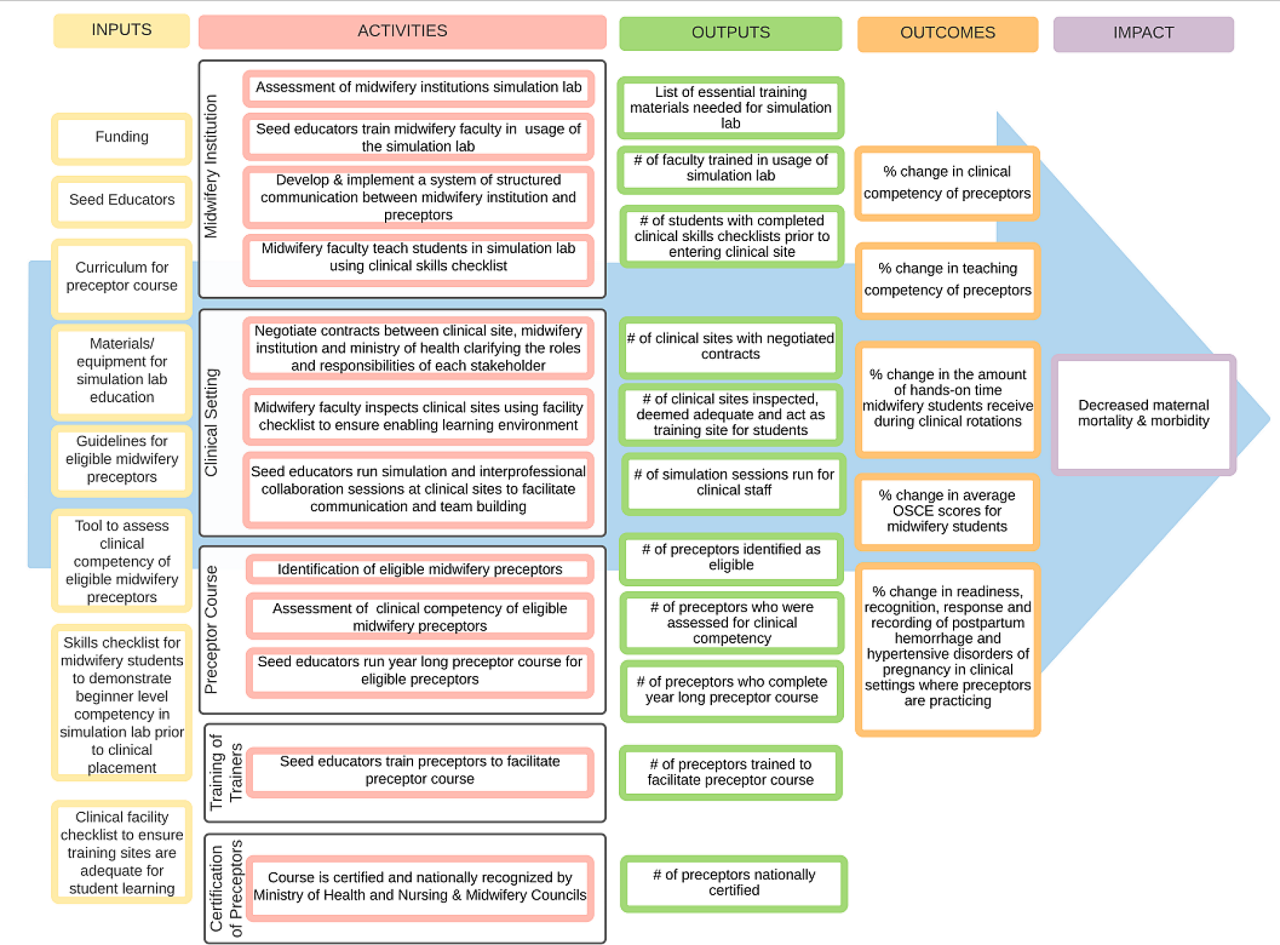
Preceptor program logic model

To be included in the Preceptor Program, minimum preceptor eligibility criteria is outlined by WHO’s *Midwifery Educator Core Competencies* for midwives, which we then adapted for Malawi nurses (i.e. qualified/licensed registered nurse, working in pediatric ward, minimum 1–2 years’ experience, demonstrate interest in teaching and educating others) [25]. All eligible candidates will be required to complete an application form and submit a letter of recommendation from a supervisor at their clinical site. A letter of recommendation from a student is also strongly encouraged. Additionally, schools may opt to conduct interviews using a rubric template to evaluate applicants. Faculty at the school and preceptor program directors will systematically and objectively review applicants and choose the most qualified candidates. We aim to recruit 25 midwifery preceptors in total across two midwifery schools in Sierra Leone to be taught in two cohorts of 10–15 each, and to recruit 15 nurse preceptors at one site in Malawi in year 1. Each following year the program is expected to grow and create impact (Table 1; Fig. 1). We also aim for a diverse body of candidates from both clinics, health centers, and hospitals.

**Table 1:** Impact over five years of proposed preceptor program in Sierra Leone and Malawi

Impact over five years of proposed preceptor program						
	Year 1	Year 2	Year 3	Year 4	Year 5	Total
**Sierra Leone (trainers)**	4 Seed educators	9 (3 Seed educators + 6 ToTs)	10 (2 Seed educators + 8 ToTs)	10 (2 Seed educators + 8 ToTs)	10 (2 Seed educator + 8 ToTs)	30 ToTs
Preceptors	20	45	50	50	50	210
Students (20 students: 1 preceptor per year)	400	900	1000	1000	1000	4200
**Malawi (trainers)**	1 Seed educator	2 (Seed educator + ToT)	2 (Seed educator + ToT)	2 (Seed educator + ToT)	2 (Seed educator + ToT)	4 ToTs
Preceptors	10	20	20	22	22	94
Students (20 students: 1 preceptor per year)	200	400	400	440	440	1880

ToT = Train-the-trainers

Total: 34 ToTs teaching the preceptor course; 304 preceptors trained; 6,080 students receiving instruction from preceptors enrolled in the program

#### Faculty and seed educators

In addition to preceptors, we will include school leadership and faculty, clinical placement leadership, management, and clinical staff (including potential preceptors), and Seed educators (visiting faculty supported by Seed Global Health, typically for 1- or 2-year contracts), to support and take part in this program. In the first year of the program, Seed educators will be the lead instructors of Step 2 activities.

### Setting

#### Sierra Leone

Currently, there are three midwifery schools, located in Freetown, Bo, and Makeni, that offer 2-year midwifery diplomas. There is one midwifery school starting in Kenema soon. Each program enrolls approximately 100 to 200 students per year. For clinical instruction, midwifery students are placed throughout the country in both urban and rural settings at community health clinics, district and regional referral hospitals. Midwives working in these various settings are expected to precept midwifery students. Yet, many of these midwives have no prior training in teaching and are offered little to no support, supervision, or compensation for their services. This study will take place at the schools of midwifery in Bo and Makeni.

#### Malawi

In 2018, there were approximately 4.4 nurses and midwives per 10,000 people in Malawi [26]. Kamuzu University of Health Sciences (KUHeS) has been training child health nurses at a master’s degree level since 2010. However, these practitioners are prepared as generalists and the program does not adequately prepare them to care for critically ill children in Malawi. For clinical instruction, nursing students are placed throughout the southern region in the central hospital, district hospitals, community hospitals and health centers. Qualified nurses working in these various settings are expected to precept nursing students; however, there is no accredited preceptor training in the country for current staff nurses who are responsible for teaching and training nursing students. This preceptor program will take place at KUHeS in Blantyre.

#### Intervention

**Fig. 2:**
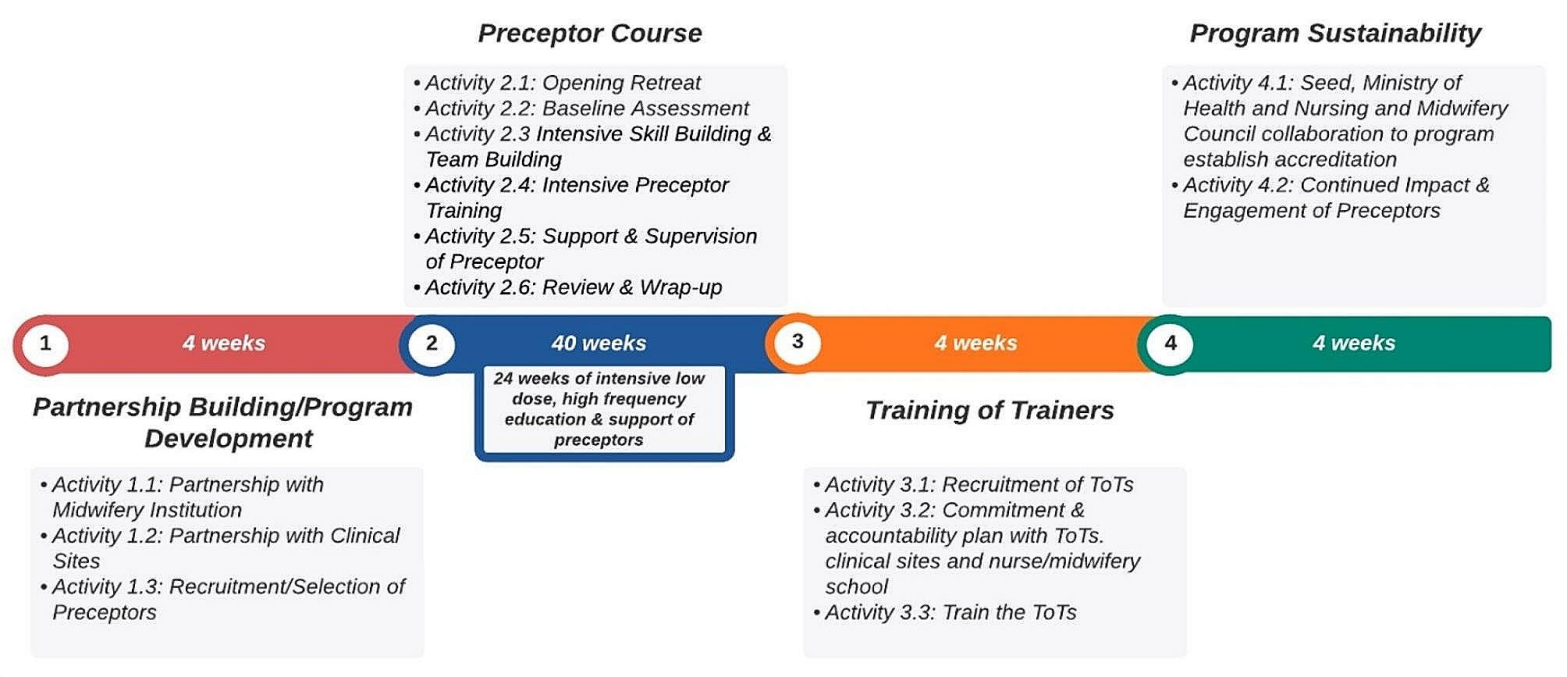
Timeline of preceptor program

The preceptor program activities aim to support and strengthen each component of the triad of schools, clinical sites, and preceptors. The preceptor program is a year-long program divided into four steps with multiple activities within each step (Table 2; Fig. 2). The intervention was developed by a team from the US, Sierra Leone, and Malawi – led by a midwife with a Master’s in Public Health (JM) and a pediatirc nurse practitioner with a PhD in nursing (BvdW). Iterative input was sought from the Seed Global Health team, an epidemiologist (for statistical support and methodological design), and partner institutions where the program will be implemented. The context and setting were central to program development and was the impetus for program development due to lack of precepting availability and high rates of maternal and child mortality, gap in theory to practice education, and partner interest. This four-step approach allows for 12 months of continuous support to ensure competency and confidence in precepting skills to ultimately support student learning via precepting in clinical facilities. This approach is also meant to limit preceptors’ time away from their clinical sites. The four steps of the program are:

**Table 2:** Preceptor program outline of activities

Step 1- Partnership Building/ Program Development (4 weeks)
Activities
**Activity 1.1** Partnership with Midwifery Institution
● Placement of Seed Educators at Institution
● Assessment of School simulation lab
● Faculty Simulation Training
● Student skills check-offs in simulation lab for students to solidify skills prior to clinical rotations
● Student orientation to clinicals before starting clinical rotation
● Development & implementation of a structured communication system between academic institution and preceptors
***Activity 1.2*** Partnership with Clinical Sites
● Clinical sites identified for Seed preceptorship & MOU developed between Clinical sites and School ● Assessment of clinical sites to assure enabling environment
***Activity 1.3***: Recruitment/Selection of Preceptors
● Request for application for Preceptors for Preceptor Program ● Selection of Preceptors jointly by Clinical Sites and Midwifery School

Step 2: Preceptor Course (40 weeks)
**Activities**
***Activity 2.1***: Opening Retreat *(2 days)*
***Activity 2.2***: Baseline Assessment *(3 weeks)*
● Assess competency and confidence in precepting students: ● Preceptor’s self-assessment ● Observation of preceptors teaching students in clinical ward
● Self-Assessment of Competencies
● Clinical Competency Pre-test
● Skills Check-off Sessions
● Simulation Sessions
● Clinical Observations
● One-on-one review/feedback session
***Activity 2.3***: Intensive Skill Building & Team Building *(12 weeks)*
***Activity 2.4***: Intensive Preceptor Training *(12 weeks)*
***Activity 2.5***: Support & Supervision of Preceptor *(7 weeks)*
***Activity 2.6***: Review & Wrap-up *(6 weeks)*
**Step 3: Training of Trainers (ToT) (4 weeks)**
**Activities**
***Activity 3.1: Recruitment of ToTs***
***Activity 3.2: Commitment & accountability plan with ToTs, clinical sites and school***
***Activity 3.3: Train the TOTs***
**Step 4: Program Sustainability (4 weeks)**
**Activities**
***Activity 4.1***: Seed, Ministry of Health and Nursing and Midwifery Council collaboration to establish accreditation
***Activity 4.2***: Continued Impact & Engagement of Preceptors

Step 1 includes partnership development, preparation of schools, clinical site(s), and preceptors, and program administration and communication logistics. Step 1 will take place over, at minimum, 4 weeks and includes three main activities: (1) partnership building with clinical sites and development of memorandums of understanding between the sites and midwifery and nursing schools, (2) assessment of clinical sites to assure they are enabling environments for students, and (3) recruitment and selection of preceptors through an application and selection process completed jointly by clinical site and schools. Ideally step 1 will only occur after a thorough needs assessment has been conducted in future locations to ensure adaptability in different settings.

Step 2 involves Seed-sponsored midwifery and nursing educators to work closely with preceptors to strengthen their evidence-based, clinical knowledge and skills and expand competence and confidence in precepting students in the clinical setting. This is done through a mixture of low-dose, high frequency didactic, simulation, and discussion-based modules and modeling of precepting over the course of 40 weeks. The 40 weeks are divided into 6 activities including: an opening retreat, baseline assessments (1 week of baseline competency and confidence self-assessment and observation of preceptors teaching students, self-assessment of competencies, clinical competency testing, skills check-off sessions, simulation sessions, clinical observation, and one-on-one review sessions between preceptors and the Seed educator), 12 weeks of intensive skill building and team building, 12 weeks of intensive preceptor training in the clinical setting, 7 weeks of support and supervision of preceptors, and 6 weeks of review and wrap-up. Throughout the preceptor program, Seed staff will have at least monthly check-in calls with implementing program facilitators to ensure program timelines, and to provide content updates and make real-time adjustments based on continuous feedback. A large focus of the program is developing a “preceptor cohort”; thus in-person learning will be emphasized. However, WhatsApp groups will be created for cohorts to communicate with each other and share resources, schedules, and to improve cohort cohesion and facilitator-preceptor relationship building.

Step 3 involves selecting trainers from the completed preceptor course and training them to implement the preceptor course for a new cohort of preceptors at their clinical site or within their region.

Step 4 involves working with the Ministry of Health and Nursing and Midwifery Councils to secure accreditation for the preceptor program, engaging and supporting the cohorts of preceptors previously trained in the program, and continuing to monitor and evaluate the effectiveness of the program. This final step is to ensure sustainability and scalability of the program.

### Data collection

#### Preceptors

During Step 2 of the preceptor program, preceptors will be given numerous pre- and post–evaluations to assess effectiveness on their competence and confidence. These evaluations will consist of validated self-assessment scales, objective structured clinical examinations (OSCEs), and direct clinical observation. Satisfaction surveys will also be given after Activity 2.1 through 2.6. Preceptors will also be invited to participate in in-depth interviews at the end of Step 2. Each scale or OSCE will take between 10 and 60 min to complete and will be administered during preceptor course time, and students’ evaluations will occur during clinical rotations.

#### Students

Students that the preceptors will precept during clinical rotations will complete written feedback surveys for the instructors.

#### Faculty, leadership, and seed educators

During step 1, faculty, leadership, and individuals who take part in launch meetings for the preceptor program will participate in process mapping activities to understand the current process of clinical precepting in their respective midwifery and nursing programs. Process mapping will elucidate facilitators and challenges to map a way forward for more effective training and skills transfer for students. Faculty and leadership involved with the program will also take brief 12-item Organizational Readiness for Implementing Change (ORIC) surveys. After Step 2, a repeat ORIC will be given and faculty, leadership and Seed educators who led Step 2 will be invited to participate in in-depth interviews.

#### Instruments

Many tools have been created or adapted for the preceptor program and are adaptable for specialties beyond midwifery and pediatric critical care (Table 3). Some validated tools will be kept in their original format. All tools will be used in English as this is the official language in both Sierra Leone and Malawi and professional healthcare and education is documented and conducted in English. From our previous work in these settings, midwives and nurses have good verbal and written English skills. The ICM Essential Competencies Self-Assessment Tool was chosen because it was developed by the international professional organization for midwives and is meant for midwives to assess their level of competence and confidence – the specific constructs we aim to measure. The tool outlines the set of knowledge, skills, and professional behaviors required by an individual to use the designation of midwife as defined by ICM when entering midwifery practice. Additionally, few tools exist to measure competence and confidence. Therefore, objective structured clinical examinations (described below) were chosen because of their common method of evaluation for student learning in the SSA setting. This is beneficial for two reasons: (1) preceptors already understand their purpose and (2) it provides an opportunity for modeling best-practice for running simulation and observed structured clinical examinations so that preceptors are more competent and confident using them in the future with students.

**Table 3:** Dashboard of data collection tools for midwifery and pediatric critical care preceptor program

Midwifery	Pediatric critical care
Participant Application	Participant Application
Interview Scoring	Organizational readiness for change survey
Checklist for simulation lab	Learning agreements with preceptors
Safe birth supplies checklist	
Baseline pre-test	Baseline knowledge pre-test
Learning agreements	NMCM/ICM adapted self-assessment of competencies

#### Process mapping

The implementation process for Step 1 will be assessed using co-created process maps with preceptor program partners during launch meetings. Processing mapping is when individuals visualize on paper the services they deliver from the perspective of the target population and identify bottlenecks and inefficiencies. Through the process map of the patient (or student) pathways, we will discuss and identify modifiable system challenges and facilitators to help modify and improve the preceptor program and overall partnership between school, clinical sites, and preceptors.

#### Organization readiness for implementing change survey

The organizational readiness for implementing change (ORIC) survey assesses the ‘extent to which organization members are psychologically and behaviorally prepared to implement organizational change [22, 27]. It is a 12-item tool assessing change commitment and change efficacy. It is important to understand leaders’ and participants’ readiness for change prior to, and during an intervention and it can help explain some reasons for program effectiveness or challenges in implementation.

Objective structured clinical examination and validated confidence and competence scales.

Objective structured clinical examinations (OSCEs) and ICM competence and confidence scales will be employed during the first three weeks of the preceptor course (Step 2) to all preceptors and again after 24 weeks of material (12 weeks of intensive skill building and 12 weeks of intensive preceptor training). This includes: preceptor’s self-assessment, observation of preceptors teaching students in clinical ward, self-assessment of midwifery competencies, a clinical competency pre-test, OSCE, group simulation sessions, clinical observations, and 1:1 feedback with the Seed educator and preceptor. ICM competencies and confidence will be measured in: general midwifery care, pre-pregnancy and antenatal care, care during labor and birth, ongoing care of women and newborns with multiple sub-scales in each domain related to knowledge, skills, and professional behaviors within each competency area. Pediatric critical care competencies and confidence will be measured in: vital signs and airway, breathing, circulation, disability, and exposure (ABCDE’s), electrocardiogram interpretation, bubble continuous positive airway pressure management, external ventricular drain management, mechanical ventilation, chest tube management, central line care, blood gas and electrolyte interpretation, and nasogastric tube insertion and feeding. These scales have been adapted from Nurses and Midwives Council of Malawi and the KUHeS Paediatric Critical Care Competencies to fit the needs of this preceptor program, specific to pediatric critical care.

#### Interviews

Semi-structured in-depth interviews will be based on the Consolidated Framework for Implementation Research 2.0 (CFIR) to guide an in-depth examination of the implementation and adoption process, define core elements of the preceptor program (versus adaptable peripheral elements), and describe determinants of success and failures across implementing sites after Step 1 and during Step 2 (Supplement 1) [28]. In-depth interviews will allow for exploration of the individual experience with disseminating and implementing the preceptor program, and capture intervention adaptations over time, such as staff attitudes or identification with the organization. All participants will be purposely selected by study personnel, to ensure balance and representation across service location and roles, and ability to speak knowledgeably about the preceptor program. We will also discuss the reach of the program and potential ways to expand and reach more sites as well as the reach towards different patient populations (i.e., beyond pregnant people, infants, and children).

### Data analysis

#### Quantitative data

To determine organizational readiness for implementing change and effectiveness of the preceptor program on preceptors’ competence and confidence, median change in 1) ORIC scores (1) OSCE scores (2) Direct Clinical Observation Scores (3) Clinical Competency test scores and (4) ICM or adapted ICM competence and confidence self-reported scales will be reported. Wilcoxon signed rank test will be used to determine if there are any significant differences pre- and post-preceptor program as outcome data are expected to be ranks (i.e., 3- and 5-point Likert scales) and not normally distributed. Analyses will be conducted using SAS version 9.4.

#### Qualitative data

In-depth interviews will occur in English, the official language in Sierra Leone and Malawi, by an experienced interviewer trained by a PhD-prepared nurse with expertise in qualitative methods and use of CFIR and implementation science [29, 30], audio-recorded, and transcribed verbatim. First, two coders in a stepwise, iterative process will code the transcripts and conduct content analysis within a deductive framework to identify key implementation and adoption themes (using selected CFIR constructs, and allowing flexibility for other themes to emerge). Coding will be compared across pairs and differences discussed prior to final coding. Case memos will be written and three analysts will assign ratings for each construct. Using a rating process previously applied to the CFIR, ratings will reflect the positive or negative influence (valence) and the strengths of each construct. CFIR constructs will be coded as missing too much data (M), not (0), weakly (+ 1/-1), or strongly (+ 2/-2) distinguishing low/high performance. Findings will be used to develop recommendations for preceptor program implementation, adoption, and increasing reach, including its core components, intervention adaptations, and lessons learned. Interviews will last approximately 30 to 60 min. All stakeholders (preceptors, facilitators, and leadership involved in the preceptor program) will be invited to participate for equity purposes even though we may reach saturation prior to completing all interviews. A small incentive for their time will be provided.

#### Parallel qualitative and quantitative design

Quantitative and qualitative data collection will occur simultaneously, and iteratively, and results will be mixed – they will be compared. Equal emphasis will be given to both forms of data during interpretation [31]. Use of data obtained by both methods will support findings to provide a more complete understanding of the preceptor program effectiveness.

#### Risk management and safety

There are minimal risks to this study. Participants may withdraw from the study at any time. Preceptors will be informed their participation is voluntary and withdrawal will not impact their job standing. Identifying information will be redacted from any surveys prior to entry into the data management system and will be redacted from transcripts during transcription.

#### Data security and handling

All data for programmatic purposes will be collected on paper and pen. Then, data will be scanned and stored on a computer with restricted access and password protected on a research drive, including consent forms and survey data. All survey data collected will be deidentified and stored on REDCap for data management. Interview data will be electronic and transcripts will be encrypted and password protected, and uploaded into Dedoose for analysis.

#### Confidentiality and security

All data will be anonymous and no individuals will be identifiable in the reporting of outcomes. Each preceptor will be given a unique identifier to match pre- and post-tests for statistical analyses. Interviews will be numbered. Any findings will be presented de-identified in journals and conference presentations.

## Discussion

This study protocol aims to assess the readiness for implementation and effectiveness of this preceptor program for midwifery and nursing in Sierra Leone and Malawi. It will evaluate the organizational readiness to implement and maintain a comprehensive preceptor program using process mapping and the ORIC survey. It will determine the effectiveness of the preceptor program on precepts’ competence and confidence after the intensive phase of the program through pre- and post-objective OSCE and validated confidence scales. Finally, it will assess the early adoption and reach of the preceptor program in Sierra Leone and Malawi institutions through qualitative in-depth interviews with institutional leaders, preceptors, and students affected by the program. Outcomes of the preceptor program will include the impact on growing a workforce of confident and competent midwife and nurse preceptors, the ability to effectively teach students in the clinical learning environment, and the creation of a new cadre of professionals in settings in need of well-trained clinician-educators. This innovative low dose, high frequency Seed preceptor program will provide a sustainable model for nursing and midwifery education and local capacity building with potential for scalability.

### Electronic supplementary material

Below is the link to the electronic supplementary material.

Supplementary Material 1

## Data Availability

Data sharing is not applicable to this article as no datasets were generated or analyzed during the current study.

## Abbreviations

CFIR: Consolidated Framework for Implementation Research 2.0
ICM: International Confederation of Midwives
ICN: International Council of Nurses
KUHeS: Kamuzu University of Health Sciences
NEPI: Nurse Education Partnership Initiative
OSCE: Objective Structured Clinical Exam
ORIC: Organizational Readiness to Implement Change
UNPF: United Nations Population Fund
WHO: World Health Organization
SSA: Sub-Saharan Africa
