# Supplementary material for: Development and initiation of a preceptor program to improve midwifery and nursing clinical education in sub-saharan Africa: protocol for a mixed methods study
Source: BMC Nurs. 2024 May 31;23:365. doi: 10.1186/s12912-024-02036-2 (PMC11141008; doi:10.1186/s12912-024-02036-2)
Supplement: Supplementary file 1 — Supplementary Material 1 [file 12912_2024_2036_MOESM1_ESM.docx]

Supplement 1 - Preceptor program post-program evaluation CFIR interview guide

**Introduction and welcome.**

Good morning/afternoon, thank you so much for agreeing to be interviewed today. My name is [X], and I’m a researcher with Boston College and Seed Global Health. We want to know about your experience in participating in and being part of the Preceptor Program. We will be recording this interview, so that later on, we can transcribe (write down) your words to learn about different people’s perspectives and bring them all together to try to understand different people’s points of views about the implementation of this program. I also might take some notes while we speak in case I want to ask more about your answers. Is it ok for us to record and take notes today? [Give time for participant to answer]. If you ever feel uncomfortable, we can stop the interview at any point.

Your answers are confidential and anonymous. We will share results in aggregate with individuals involved with the program, but will remove any identifying information. Do you have any questions before we start?

Thank you.

**Demographic questions**

Gender:

Age:

Years as a nurse:
Title at work (i.e. matron, head nurse, charge nurse):

Ward/unit you work on (i.e. pediatric HDU, PICU, general pediatrics, nutrition, etc):

OVERALL AIMS:

- What has **facilitated** the implementation of this preceptor program and what have the **barriers** been to implementing it? (Thinking back)
- Where do you **envision the role of the preceptor** going or being absorbed in your work setting/organization? (Thinking forward)

1. In thinking about the advantage, needs, or resources of those served by your organization(s), how does the preceptor program compare to other similar or existing programs in your setting?
   1. What are the advantages of the preceptor program compared to existing programs (if any)?
   2. What are the disadvantages?
   3. Is there another intervention (as opposed to the preceptor program) that people would rather implement (to improve pediatric critical care/midwifery)?
      1. Can you describe that intervention?
      2. Why would people prefer the alternative?
2. How complicated was the preceptor program?
   1. For example, the duration, scope, intricacy and number of steps involved?
   2. Did the intervention (preceptor program) reflect a clear departure from previous practice (i.e. nurses providing clinical supervision/precepting without formal precepting training/education)?
3. What is your perception about the likelihood of this preceptor program expanding so that another preceptor program could be available across a wider geographic or additional practice settings?
4. What is your perception about the likelihood of continued use of program components and activities for the continued achievement of desirable preceptor program and population outcomes (i.e. more pediatric critical care preceptors and improved child health outcomes)?
   1. What type of resources will you need for continuity?
5. How “in touch” are staff and leadership with the individuals served by your organization (Seed/KUHeS)?
6. How well do you think the preceptor program will meet the needs of the individuals served by your organization? (KUHeS, students/Queens/patients)?
7. How will the infrastructure of your organization (social architecture, age, maturity, etc) affect the implementation of the intervention (preceptor program)?
   1. How will the infrastructure facilitate/hinder implementation of the preceptor program?
   2. How will preceptors be utilized moving forward? How will they be absorbed into the existing system?
8. What kinds of infrastructure changes will be needed to accommodate the intervention (preceptor program)?
   1. Change sin scope of practice? Changes in formal policies?
   2. What kinds of approvals will be needed? Who will need to be involved?
   3. How will preceptors be utilized moving forward?
9. How do you think your organization’s culture (general beliefs, values, assumptions that people embrace) will affect the implementation of the intervention (preceptor program)?
10. What is the general level of receptivity in your organization to implementing the intervention (preceptor program)?
11. Is there a strong need for this intervention? Why/why not?
12. How essential is this intervention to meet the needs of the individuals served by your organization or other organizational goals and objectives?
13. How do people feel about current programs/practice/process that is happening related to the intervention (preceptor program)?
    1. Current precepting that happens on wards and the relationship/interactions between university and hospital settings, or lecturers and staff nurses
14. What kinds of high-priority initiative or activities are already happening in your setting?
    1. How do we prioritize teaching students and precepting for quality healthcare (and saving children’s lives)?
15. What kinds of incentives are there to help ensure that the implementation of the intervention is successful?
    1. What is your internal AND external motivation for wanting to help ensure the implementation is successful?
16. To what extent do you think your supervisor will consider your role in this implementation in regard for your work or role?
17. To what extent do you feel like you can try new things to improve your work processes?
    1. Do you feel like you have the time and energy to think about ways to improve things?
18. What level of involvement has leadership at your organization had so far with the intervention?
19. What kind of support or actives can you expect from leaders in your organization to help make implementation successful?
    1. Who are these leaders? How do attitudes of different leaders vary?
    2. Do they know about the intention to implement the intervention?
20. Do you expect to have sufficient resources to implement and administer the intervention (preceptor program/precepting)?
    1. What resources are you counting on? Are there any resources that you would have like to received?
21. Who do you ask if you have questions about the intervention or its implementation? How available are these individuals?
22. Do you think the intervention is effective in your setting? Why/why not?
23. At what stage of implementation is the intervention (precepting in general/what you’ve learned in the program) at your organization?
    1. How do you think the program (precepting in general) is going? Why?
24. How confident are you that you will be able to successfully implement the intervention (precepting in general/what you’ve learned in the program)?
    1. What gives you that confidence/lack of confidence?
25. Other than the formal implementation leader, are there people in your organization who are likely to champion (go above and beyond what might be expected) the intervention?
    1. Were they formally appointed in this position or was it an informal role?
    2. What position do these champions have?
    3. What kinds of behaviors or actions do these champions exhibit? (For example, helping get senior leaders on board, helping solve problems? Or a small role?)
26. Has the intervention been implemented according to the implementation plan that you were made aware of? Can you describe this?
27. Is there anything else you would like to share with us that we haven’t talked about today?

Thank you so much for your time.
